# A facilitated social innovation: stakeholder groups using Plan-Do-Study-Act cycles for perinatal health across levels of the health system in Cao Bang province, Vietnam

**DOI:** 10.1186/s43058-023-00403-9

**Published:** 2023-03-10

**Authors:** Anna Bergström, Dinh Phuong Hoa, Nguyen Thu Nga, Trieu Hoa, Tran Thanh Tu, Pham Thi Lan Lien, Tran Trang, Lars Wallin, Lars-Åke Persson, Leif Eriksson

**Affiliations:** 1 SWEDESD, Department of Women’s and Children’s Health, Uppsala University, Uppsala, Sweden; 2 Institute for Global Health, University College London, London, UK; 3 Research Institute for Child Health, Vietnam National Children’s Hospital, Hanoi, Vietnam; 4 Hanoi University of Public Health, Hanoi, Vietnam; 5 Provincial Reproductive Health Bureau, Provincial Health Bureau, Cao Bang, Vietnam; 6 School of Education, Health, and Social Studies, Dalarna University, Falun, Sweden; 7 London School of Hygiene & Tropical Medicine, London, UK; 8 Department of Public Health and Caring Science, Uppsala University, Uppsala, Sweden

**Keywords:** i-PARIHS, Implementation science, Knowledge translation, Social innovation, Facilitation, PDSA cycles, Vietnam, Perinatal health

## Abstract

**Background:**

Universal coverage of evidence-based interventions for perinatal health, often part of evidence-based guidelines, could prevent most perinatal deaths, particularly if entire communities were engaged in the implementation. Social innovations may provide creative solutions to the implementation of evidence-based guidelines, but successful use of social innovations relies on the engagement of communities and health system actors. This proof-of-concept study aimed to assess whether an earlier successful social innovation for improved neonatal survival that employed regular facilitated Plan-Do-Study-Act meetings on the commune level was feasible and acceptable when implemented on multiple levels of the health system (52 health units) and resulted in actions with plausibly favourable effects on perinatal health and survival in Cao Bang province, northern Vietnam.

**Methods:**

The Integrated Promoting Action on Research Implementation in Health Services (i-PARIHS) framework guided the implementation and evaluation of the Perinatal Knowledge-Into-Practice (PeriKIP) project. Data collection included facilitators’ diaries, health workers’ knowledge on perinatal care, structured observations of antenatal care, focus group discussions with facilitators, their mentors and representatives of different actors of the initiated stakeholder groups and an individual interview with the Reproductive Health Centre director. Clinical experts assessed the relevance of the identified problems and actions taken based on facilitators’ diaries. Descriptive statistics included proportions, means, and *t*-tests for the knowledge assessment and observations. Qualitative data were analysed by content analysis.

**Results:**

The social innovation resulted in the identification of about 500 relevant problems. Also, 75% of planned actions to overcome prioritised problems were undertaken, results presented and a plan for new actions to achieve the group’s goals to enhance perinatal health. The facilitators had significant roles, ensuring that the stakeholder groups were established based on principles of mutual respect. Overall, the knowledge of perinatal health and performance of antenatal care improved over the intervention period.

**Conclusions:**

The establishment of facilitated local stakeholder groups can remedy the need for tailored interventions and grassroots involvement in perinatal health and provide a scalable structure for focused efforts to reduce preventable deaths and promote health and well-being.

**Supplementary Information:**

The online version contains supplementary material available at 10.1186/s43058-023-00403-9.

Contributions to the literature

- Social innovations could provide creative solutions to address the implementation of evidence-based guidelines and healthcare delivery challenges for perinatal health.
- Implementation of regular facilitated meetings with stakeholders applying a structured process for undertaking tailored actions to locally identified problems and using available evidence-based guidelines ensured end-users involvement and development of a needs-based and responsive health system for perinatal health.
- Engaging communities and health system actors across different sectors of society in structured processes aiming to improve health and survival is a promising example of the enabling environments that the global 2030 agenda for women and children health and survival set out to achieve.
- The article presents a way to assess the relevance of identified problems alongside their coupled goals and actions and the completeness of the application of the steps in the PDSA cycle and provides a more accurate understanding of the actual use of the PDSA cycle.

## Background

The period with the most wide-ranging consequences for short- and long-term health and well-being is before and during delivery and the first 4 weeks after delivery, i.e. the perinatal period [1]. Despite the major progress during the last few decades, there are annually more than 1 million intrapartum-related stillbirths worldwide, 2.5 million deaths within the first month of life and around 250,000 maternal deaths [2]. Within global perinatal health, increased quality and coverage of a series of simple, evidence-based and cost-effective practices can avert 70% of the neonatal deaths, 30% of the stillbirths caused during delivery (intrapartum) and 50% of maternal deaths [3, 4]. The most crucial time for these evidence-based practices is during delivery and within the first week of life [5]. With the Sustainable Development Goals launch, the global community has committed to ending preventable deaths of newborns and children under 5 years of age and ambitiously reducing maternal deaths by 2030 [6]. With its direct and indirect effects on health, the provision of health services and economy, the current pandemic threatens the maternal and child health gains of the last decades [7]. There is now momentum to identify approaches to systematically fulfil these ambitions and provide evidence on how governments can implement these at scale.

Social innovations are usually seen ‘as a set of processes that amalgamate combinations of up to three related propositions’: new forms of collaboration, restructuring of social or power relations, and positive societal impact [8]. Social innovations in health generally include community participation, engaging multiple stakeholders, being needs-based and contributing to transformation in health and wellbeing [9]. Community engagement is an essential factor driving this culture shift. Despite a growing body of evidence on the potential impact of community engagement [10, 11], maternal and child health services are often implemented without the end-users involvement [12]. The result is a lack of trust and underutilisation of health services, inequity in care utilisation and stagnating health indicators*.* When (re)building post-pandemic maternal and child health, these aspects need consideration [7]*.*

In implementation science, *facilitation* is described as a social approach to increase the use of evidence-based methods [13]. Facilitation implies having a person who enables others to implement a practice change with an emphasis on *supporting* rather than *prescribing* [14]. Facilitation is commonly coupled with other social innovations, e.g. the Plan-Do-Study-Act (PDSA)—a powerful bottom-up approach that includes identifying and prioritising a locally identified problem followed by strategic action and careful monitoring.

The Newborn Knowledge Into Practice (NeoKIP) project in the Quang Ninh province, Vietnam, resulted in reducing neonatal mortality by half (OR_adj_ 0.51; 95% CI 0.30–0.89) [15]. The social innovation tested implied that local stakeholder groups, composed of primary care staff, local politicians and representatives of the local Women’s Union, applied the PDSA approach to locally identified problems. Trained facilitators recruited from the Women’s Union, a well-established nationwide organisation, supported the local stakeholder groups. In addition to reducing the neonatal mortality rate, this social innovation also increased attendance to antenatal care [15] and increased equity in neonatal survival [16]. In contrast to previous women’s group studies [17], the NeoKIP innovation focused on initiating groups consisting of local healthcare staff and local stakeholders, i.e. trained professionals and influential commune members. The rationale for establishing this type of group was the feasibility of scaling up the innovation nationally if having sustained effect. This social innovation was primarily built upon local ownership and empowerment of the local stakeholders at the lowest hierarchical administrative and health system level. As expected, the implementation of the innovation did not result in an immediate effect on neonatal mortality. Instead, a delay in the effect was anticipated [15, 18–20]. In NeoKIP, each stakeholder group met with their facilitator monthly over three years (2008–2011). In 2014, a follow-up survey was conducted in the Quang Ninh province, 3 years after completing the trial. A sustained low level of neonatal mortality rate was detected in the intervention communes [21]. Like in other community-based strategies, the NeoKIP innovation did not show a reduction in stillbirths or intrapartum-related neonatal deaths, which requires improved quality of care at the hospital level [22]. This fact motivated future participatory interventions to be implemented at both the primary care level and the secondary (hospital) level to improve the quality of perinatal care provided. This need has also been expressed in a review of participatory action trials [17]. Furthermore, the WHO recommends such approaches for improved perinatal health [23].

## Guiding theoretical framework

**Fig. 1:**
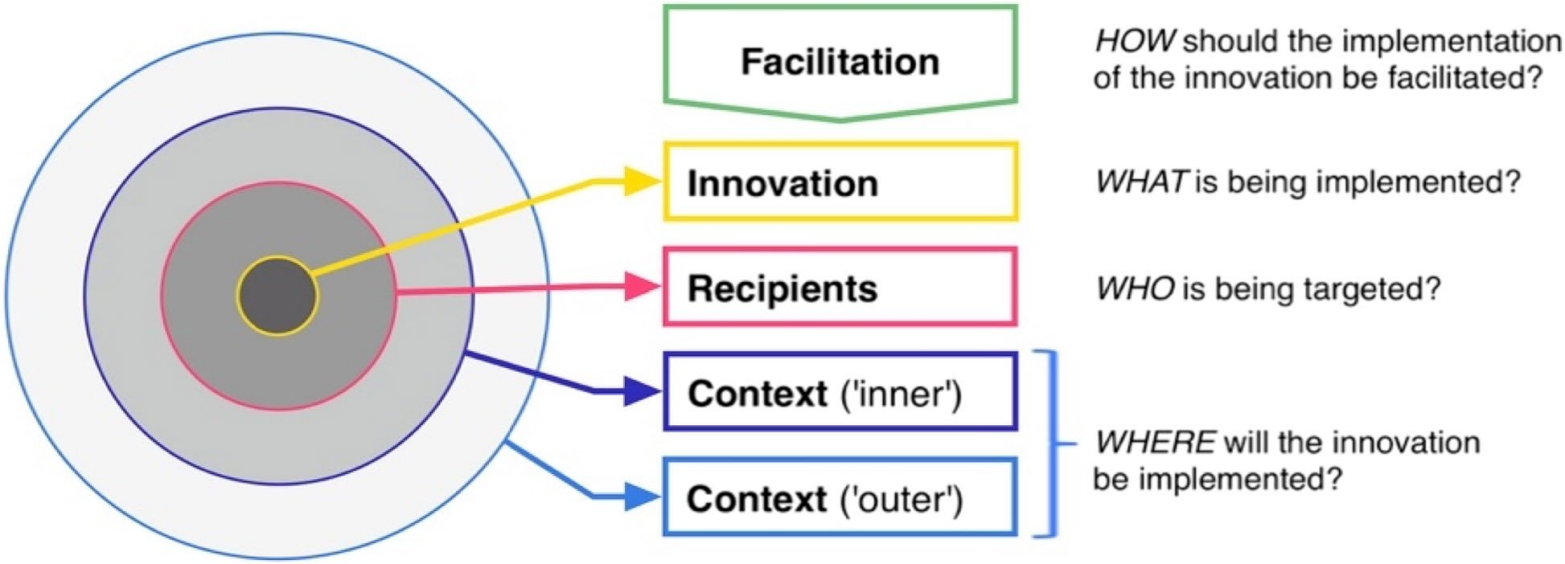
The i-PARIHS framework

The current project builds upon the Integrated-Promoting Action on Research Implementation in Health Services (i-PARIHS) framework, which posits the interplay between the *innovation* to be implemented, the *recipients* who will either use the innovation or are targeted by the innovation (sometimes also referred to as ‘end-users’) and the *context* in which the innovation is implemented [24]*.* The i-PARIHS presents *facilitation* as the ingredient that activates the implementation through assessing and responding to the characteristics of the other three components [24] (Fig. 1). The current project evaluates a social innovation where *facilitation* functions to support the implementation of systematic improvement (*innovation*) through local stakeholder groups (*recipients*) on the primary, secondary and tertiary healthcare levels (*context*).

### Facilitation

**Fig. 2:**
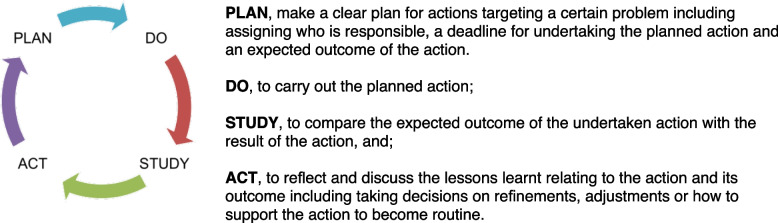
The Plan-Do-Study-Act cycle

A successful implementation constitutes the four elements in the i-PARIHS framework [24]. A facilitator has an essential role in knowledge translation, i.e. to assist teams in apprehending what needs to be changed in their local setting and facilitate the process where the teams identify how it can be changed [14, 25]. The facilitator thereby holds an important position to achieve successful implementation. Recent reviews of facilitation have unanimously concluded that it is a promising approach for translating evidence into practice [14, 17, 26, 27]. One commonly used method often coupled with facilitation is the action-oriented PDSA cycle [28]. The PDSA cycle prescribes a four-stage cyclic learning approach to adapt changes aimed at improvement (Fig. 2).

The pragmatic principles of PDSA cycles promote the use of a small-scale, iterative approach to test interventions or actions, enabling rapid assessment and providing flexibility to adapt changes to ensure fit-for-purpose solutions [29, 30]. The characteristics of the PDSA is likely influencing its ability to diffuse into health systems in high-, middle- and low-income settings [28]. Based on the current evidence, the WHO recommends implementing facilitated PDSA on health facility and community levels for improving maternal and newborn health [23, 31, 32]. The PDSA approach can easily be integrated into systems and become a sustainable continuous learning and action method.

This study aimed to assess whether an earlier successful social innovation for improved neonatal survival that employed a PDSA approach on the commune level was feasible and acceptable also when implemented on multiple levels of the health system and resulted in actions with plausibly favourable effects on perinatal health and survival. Furthermore, this project aimed to get better insights into how different stakeholders perceived that social innovation.

## Methods

### Study setting

The Perinatal Knowledge-Into-Practice (PeriKIP) project was undertaken in Cao Bang province, situated in the northern part of Vietnam bordering China. Cao Bang province has about 520,000 inhabitants [33]. The province consists of one town municipality and 12 rural districts and has 12 district hospitals and one provincial hospital. The province includes impoverished areas with a high proportion (94%) of inhabitants belonging to ethnic minority groups [34] that have higher neonatal mortality than the majority [35]. At the time of initial project discussions in 2014, the official neonatal mortality rate (NMR) in Cao Bang province was 14 death per 1000 live births. However, official data on neonatal mortality in Vietnam has earlier been underestimated [36]. Based on discussions between the research team, the Reproductive Health Centre, and the Provincial Health Bureau in Cao Bang, three districts were purposively selected based on unfavourable characteristics regarding neonatal deaths, geographical location, and proportion of home-based deliveries.

The Vietnamese provincial health system has three levels: (I) the primary healthcare at commune health centres, (II) the secondary referral level at the district hospitals and (III) the tertiary care level at provincial hospitals. Emergency and comprehensive obstetric services and inpatient care are provided at district or provincial-level hospitals. Commune health centres provide primary healthcare services, such as normal birth assistance, basic obstetric care and outpatient care [37]. The PeriKIP interventions were implemented in 52 health units: at 48 commune health centres from November 1, 2015, to October 31, 2016, and at three district hospitals and the provincial hospital from November 1, 2015, to April 30, 2017. The rationale for running the intervention on the hospital level 6 months longer was the perceived need of understanding the functionality of the social innovation on this higher level of care.

### Description of the social innovation

The basic feature of the PeriKIP social innovation was that trained facilitators supported local stakeholder groups at the commune level and at district and provincial hospital levels in their efforts to improve perinatal healthcare practices. Seven laywomen from the Women’s Union were recruited as facilitators on the commune level. Facilitator positions were advertised openly, and recruitment was based on applicants’ previous experience with community activities and communication skills. A retired director (physician) of the Reproductive Health Centre on the provincial level in Cao Bang was recruited and trained to take the role as facilitator in the participating four hospitals. The project was implemented within the existing healthcare system [37] to increase the local accountability and ownership of quality improvement among stakeholders responsible for health (see Table 1). The PeriKIP groups at the three different levels were expected to meet once a month for the project’s duration. Participating in meetings and actions within PeriKIP was expected to be part of the stakeholders’ duties. Therefore, none was paid for their engagement besides the village health worker and the Women’s Union worker from the village level, who were reimbursed for travel expenses enabling them to attend monthly meetings.

**Table 1:** PeriKIP group stakeholders at three health system levels

*Stakeholder groups at the commune level*: Each commune has one Commune Health Centre providing primary healthcare. In each of the communes in the study area (*n*=48), one PeriKIP group was established with the following eight participants: three Commune Health Centre staff (head of Community Health Centre, midwife and nurse), one village health worker, one vice chairperson of the Peoples committee, one women union representative from community level, one women union representative from village level and one population officer
*District and provincial hospital level*: In each of the district hospitals in the study area (*n*=3) and in the provincial hospital (*n*=1), one PeriKIP group was established with the following eight participants: one midwife from the antenatal care clinic, one midwife from the labour ward, the head nurse of the paediatric department, the head of the obstetric department (physician), the head of the paediatric department (physician), the head of the general planning department, the leader of the hospital director board and one representative from Reproductive Health Centre at district or provincial level

During 2 weeks, the research group trained locally recruited facilitators with theoretical sessions, group discussions and role-play activities. Topics covered group dynamics and quality improvement methods (brainstorming and the PDSA cycle). To facilitate discussions about perinatal care, the facilitators were introduced to basic evidence-based neonatal care per recommendations in the Vietnamese National Guidelines in Reproductive Health Care [38]. Also, facilitators were briefed on the current health situation in their respective districts and the function of the healthcare system concerning reproductive health. Guides on facilitators’ roles, attitudes, responsibilities and how to handle challenging situations were based on the i-PARIHS framework [24] and modified materials from the NeoKIP project [39]. At the end of the training, facilitators practised their skills in rural communes and district hospitals outside the study area followed by feedback discussions on performance. One person with reproductive health responsibilities from each district was recruited as a mentor of the facilitators working in the communes of that district. These persons attended the facilitator training and participated in separate sessions focusing on how to mentor facilitators. A guide describing the role of the mentors was also developed and used to support the mentors in their roles. Members of the research group were not involved in delivering the intervention to the local stakeholder groups. Trained facilitators within PeriKIP received a monthly salary.

### Data collection

This study employed a convergent mixed-methods study design [40]. Quantitative and qualitative data were collected concurrently on the process and outcomes of the social innovation. The results are presented separately but discussed together in the discussion.

#### Process evaluation and outcomes

A process evaluation employing qualitative and quantitative methods prospectively tracked the implementation to describe how the social innovation was initiated, carried out and how participants responded to the innovation. The process evaluation was performed according to the UK Medical Research Council guidelines: implementation, mechanism of impact and context [41]. We developed a logic model, underpinning the assumptions on which the intervention was thought to function (see Additional file 1). For each of the three components, key questions needing an answer to understand the process were formulated, followed by identifying the target population, data sources, procedures, and tools (see Additional file 1). *The implementation of the innovation*: What was delivered and how it was delivered, including the procedures used to approach and attract facilitators, mentors, and group stakeholders (*recruitment*), the participation (*reach*), and the efforts of the facilitators (*dose*). *Mechanism of impact*: The participants’ responses to and interactions with the innovation. In this component, we explored why specific reactions to social innovation resulted in particular outcomes. Furthermore, we also explored the problems the groups addressed, the type and relevance of prioritised issues, actions taken, the interaction between group and facilitator and methods used. *Context*: What contextual aspects that influenced the innovation, the implementation and the mechanism of impact, leading to different outcomes. The *outcomes* of the social innovation included the relevance of identified problems and completion of PDSA cycles, knowledge of perinatal care, perspectives of gaining knowledge and performance of antenatal care. The following data collection modes and tools were used to monitor data of the three process evaluation components and the outcomes of the social innovation:

##### The facilitators’ diaries

The facilitators’ diaries contained sections to gather data about the stakeholder groups’ adherence to the PDSA approach (i.e. completed full cycles or not) and information about stakeholders’ attendance, problems identified, why problems were prioritised, actions planned to address these problems and whether actions were implemented as planned. The facilitators made notes in the diaries after each stakeholder group meeting.

##### Knowledge assessment

A questionnaire with 22 questions (see Additional file 2) for assessing staff knowledge on definitions of the perinatal and neonatal period (*n* = 2), antenatal care (*n* = 7) and postpartum care (*n* = 13) was developed based on the National Guidelines in Reproductive Health Care [38] and administered to health workers involved in the provision of care to pregnant and birthing women, newly delivered women and their newborns at the onset of the study and the end of the 12-month intervention.

##### Provision of antenatal care at the commune level

An observational checklist (see Additional file 3) for routine antenatal care visits was developed. It captured eight recommendations in the National Guidelines in Reproductive Health Care [38] and included 81 items (see Table 2). Assessments were made using a scale with the options yes/no/don’t know/cannot observe. Midwifery teachers were trained to undertake structured and non-participatory observations of four women’s antenatal care visits in each commune health centre at the beginning and the end of the PeriKIP project.

**Table 2:** Domains covered in the observational checklist

1. Asking questions about the pregnant woman’s health and reproductive health history
2. General examination of the pregnant woman
3. Performing different tests
4. Tetanus vaccination (counselling and provision of vaccines)
5. Provision of essential drugs
6. Health education for pregnant women
7. Recording patient information
8. Danger signs during pregnancy, birth preparedness and feedback on examinations

##### Qualitative data collection

Facilitators participated in focus group discussions (FGDs) after the training, 6 months later and after completing the project. A total of 15 FDGs with homogeneous groups of PeriKIP stakeholders from the commune level were undertaken in each district after 12 months: village health workers (*n* = 3), vice-chairpersons (*n* = 3), midwives (*n* = 3), commune health centre heads (*n* = 3) and Women’s Union representatives (*n* = 3). The rationale for undertaking FGDs with homogeneous groups was to understand how different stakeholders perceived their roles in the groups, allowing for potentially critical comments about other stakeholders’ involvement. One FGD was undertaken with each of the PeriKIP groups at the hospital level (*n* = 4) and one with the three mentors. Lastly, one individual interview was undertaken with the Reproductive Health Centre director in Cao Bang province after 12 months of implementation. The qualitative data collection aimed at understanding the mechanisms of change: the acceptability, usefulness, and operationalisation of the intervention at different levels [42, 43]. The question guides were inspired by the i-PARIHS [44] and the UK Medical Research Council framework [41]. The FGDs and the interview were audio-recorded and lasted 60–90 min.

### Data analysis

Two pairs in the research team (one neonatologist and one paediatrician in the first pair, one paediatrician and one general physician in the other pair) analysed the relevance of the identified problems and actions taken based on facilitators’ diaries from the 52 facilities. The independent scoring of each analyst was discussed to reach a consensus. A maximum score of 22 points could be obtained in the knowledge assessment (1 point for each correctly answered question). Baseline and endline results were compared across levels of the health systems (commune health centres and hospitals) and categories of health workers (physician, midwife and nurse). Data from the antenatal care observations were compared before and after the 12 months of PeriKIP intervention. Data from knowledge assessment and observations were entered using EpiData (version 3.1) and analysed in SAS (version 9.4). Descriptive statistics included proportions, means with 95% confidence intervals and *t*-tests with *p*-values.

The qualitative data were transcribed verbatim, translated into English and analysed by content analysis with both inductive and deductive features [45]. First, each interview of each type (midwives, village health workers, PeriKIP groups at hospitals, facilitators) was read several times to get a naïve understanding. This step informed the decision to approach the material as one data set. After that, open coding was undertaken. Codes were written in the margin of each interview describing aspects of the content. Codes were sorted into sub-categories; thereafter, sub-categories were sorted under categories, and finally, categories were placed under four main categories, i.e. the i-PARIHS dimensions (Innovation, Recipients, Facilitation and Context). One relevant category, *Gaining knowledge and insights*, as identified in the qualitative analysis, could not be sorted under the i-PARIHS dimensions. This category is presented together with the study outcomes.

## Results

The findings are presented as *Implementation* (reach and dose), *Mechanisms of impact* and *Context* (quality of facilitation, of the social innovation and the context in which it was implemented) and *Outcomes* (relevance of the identified problems and completion of the PDSA cycle, understanding of perinatal health issues, antenatal care practice and accountability of quality improvement processes).

### Implementation

#### Reach

##### Launching

The Reproductive Health Centre at the provincial and district levels organised launching to introduce PeriKIP and create buy-in at provincial and district levels. On the provincial level, there was representation from the Provincial Health Bureau and the provincial-level Reproductive Health Centre as well as district-level leadership representation from the Population committee, the Women’s Union and the hospital leadership from each of the included districts. At the launching of the PeriKIP on the district level, there were representatives from the commune level, i.e. representation from the population committees (Nguyên Bình 16/20; Hà Quảng 17/19; Phục Hòa 9/9), Women’s Union (Nguyên Bình 18/20; Hà Quảng 14/19; Phục Hòa 8/9) and the Commune health centre (Nguyên Bình 20/20; Hà Quảng 19/19; Phục Hòa 9/9), the district level including representation form the district hospitals, the District Health Bureau and the District-level Reproductive Health Centres as well as representation from the provincial-level Reproductive Health Centres.

##### Attendance to PeriKIP meetings

In total, 12 meetings were held in each commune (total of 576 meetings across all included communes) and 18 meetings at each hospital (total of 72 meetings across all included hospitals). Overall, the mean attendance of different representatives at the PeriKIP group meetings was 95% (range 86–100%) (Table 3).

**Table 3:** Attendance to monthly PeriKIP meetings

**Attendance to monthly meetings per stakeholder category**		
**Commune-level PeriKIP groups (*n* = 48)**	Head, commune health centres	96%
	Midwives	96%
	Vice-chairperson	91%
	Village health worker representatives	98%
	Nurses	97%
	Population officers	94%
	Women’s Union representatives (community level)	93%
	Women’s Union representatives (village level)	95%
**Hospital-level PeriKIP groups (*n* = 4)**	Midwives working in antenatal care	99%
	Midwives working in the labour ward	99%
	Head nurses of the paediatric departments	94%
	Head doctors of the obstetric departments	94%
	Head doctors of the paediatric departments	99%
	Heads of general planning departments	96%
	Representatives from the hospital director boards	100%
	Representatives from the reproductive health centre at the district or provincial level	86%

### Mechanisms of impact and context

In total, the 48 communes identified and initiated actions to tackle 416 problems during 12 months, whilst the four included hospitals identified and initiated actions targeting 88 problems during 18 months. Below, we present the qualitative findings using i-PARIHS’ dimensions as main categories (Table 4).

**Table 4:** Qualitative findings using i-PARIHS dimensions as main categories organising categories and sub-categories

**Innovation**	**Recipients**	**Facilitation**	**Context**
**Initiation and development of PeriKIP meetings** **Functionality and purpose of the social innovation** • Apprehending the aim with the project • The composition of the group • Role of the stakeholders • Qualities of the social innovation **Using the PDSA model** • Understanding the PDSA model • Identifying and prioritising problems • Using evidence **Sustainability of the social innovation**	**Recipients’ engagement in the social innovation** **Challenging situations**	**Recruitment of facilitators and mentors** **Motivation to become a facilitator** **Becoming an experienced facilitator** • Training of facilitators and mentors • Development in the facilitator role **Being a facilitator** • Enabling successful meetings • Strategies and tasks • Handling challenges • Reflections on the functionality of the facilitation **Mentoring**	**Resources** **Traditions and norms** **Geography** **Problems reaching groups**

#### The innovation

The initiation of PeriKIP meetings was positively influenced by the careful anchoring of social innovation at the Provincial Health Bureau and the Reproductive Health Centre at the provincial and district levels. All communes and hospitals were informed about PeriKIP through these formal channels. At the beginning of the implementation phase, i.e. the first 2–3 months, the facilitators were tense and many stakeholders found the meetings’ intention unclear. Some feared social innovation to be a way for higher levels to exercise control.

To tell you the truth, everyone including our facilitator and her mentor did not know what to do in the first two meetings. Therefore, in the first two meetings, everyone, even the directors, thought that the project aimed to identify our shortcomings. For that reason, everyone in the stakeholder group presented our successes. Nobody dared to talk about our shortcomings. Doctor, stakeholder group at hospital level

The facilitator and the mentor spent time developing a trustful relationship. The groups initially focused on relatively limited and achievable problems. By the end of the project, all stakeholders could explain the purpose of the innovation and clearly describe the different steps of the PDSA cycle. The cycle provided an opportunity for everyone to contribute to the discussion, a structured way to review the current practices and a method allowing participants to register improvements and their impact.

With the working method of PeriKIP we have learnt different lessons after each meeting; for instance, we know what and how much we have done comparing to the target that we had set. We have of course done communication programs, previously but we didn’t evaluate the month’s work. Within this project, we access the result right away. That is something new. Commune health center head, stakeholder group at commune level

Overall, the social innovation was perceived as useful, and the Plan and Study steps provided a structure for the work. Previously, many activities had been initiated, but neither perceived to be adapted to the local context nor appropriately evaluated. The groups were encouraged by improvements that happened because of the groups’ actions. Although many stakeholders had attended a range of meetings at the commune health centre or hospital in the past, they perceived PeriKIP meetings to be different. The main reason was that the facilitator encouraged participation by all stakeholders, whereas previous meetings focused on passive reporting. Initially, the groups moved slowly and stakeholders were quiet, but later, the teamwork improved and all stakeholders could share ideas and became friendly and confident to participate in the discussion.

*I don’t know how to describe it. The facilitator helped to unbutton the point we get stuck in.* Village health worker, stakeholder group at commune level

Overall, the stakeholders perceived their inclusion as key to achieving the objective of PeriKIP. Some thought the activities could be improved with more stakeholders in the group, e.g. having representatives from all villages in the commune-level groups. Others suggested additional stakeholders, e.g. representatives from the Youth Union that could contact newly married couples, or from the Farmers Union that could bring up issues of food security and nutrition but also be a communication channel to their members to ensure that perinatal health and survival to be on the agenda also in other sectors of society.

Stakeholders could clearly articulate the reason for their involvement and provide a rationale for the participation of others. It was clear that they acknowledged the different stakeholders of the group as experts in different areas. Local authorities’ engagement was commonly mentioned as a prerequisite for success, including the authorisation given by the district health bureau.

The Commune health center is not enough, I think that we [the Women’s Union] play an influential role in working with the program [PeriKIP]. We impact on women in the period before childbirth, pregnancy check, iron supplementation, nutrition, for example. The later period, 1 or 2 months after delivering, the medical part can help with specializations, how to take care of newborns, what and how to feed the babies. It’s effective that way. Women Union representative, stakeholder group at commune level

PeriKIP stakeholders identified problems based on their understanding of the local situation and by using available statistics. Issues were prioritised based on the feasibility of finding a solution (time, skills, knowledge, funding and other resources), the urgency or severity of the outcome and commonality. If stakeholders disagreed, they voted. Some villages in the same commune had different problems, why groups opted to address those in parts of their commune. The stakeholders listed resources used for action, e.g. materials used when sensitising community members. They would also ask facilitators to help find new knowledge or act as a bridge to the Reproductive Health Centre’s resources.

*We used new materials, and the book on health care provided by the program.* Commune health center head, stakeholder group at commune level

The opinions varied on whether the social innovation should be integrated into routine practice. The participants articulated a fundamental understanding of the PDSA process, including all its steps, recognised the benefits of engaging different stakeholders and recognised an external facilitator’s role as catalyst. The perception was that the facilitator had other skills than the PeriKIP group stakeholders, and the fact that a person came to the commune solely for this purpose gave weight to the meetings. However, several suggested that this activity could be integrated into other arrangements, e.g. the commune health centre’s meetings with all village health workers. As such, the social innovation and the PDSA model were seen as a way of working that could be sustained at both the community and hospital levels, maybe in a slightly different format.

After one year the members of the group have become used to this working procedure and therefore I and the department will stick to its objectives and working steps to improve ourselves even when PeriKIP comes to an end. Doctor, stakeholder group at hospital level

#### Recipients

The PeriKIP group environment was inclusive with an active engagement in discussions. This characterisation was brought forward as a critical trait of the groups. The monthly meetings were fora for dialogue, mutual learning and formulating common goals.

Both facilitators and stakeholders recognised that the social innovation depended on all contributing to the group’s work and ensuring accountability of the activities. All stakeholders considered themselves and found other stakeholders to be essential for the group. The vice-chairperson stood out as a decision-maker in the commune groups, and in the hospital, one of the administrative heads had that role. The participation of these individuals was indispensable for the facilitators.

The representative for village WU for example, they took part in the discussion, they shared that the rate of home birth was very high. That I couldn’t have found out myself. Moreover, this made me and them closer. Commune health center head, stakeholder group at commune level

The understanding of perinatal health problems varied within the groups and over time. Some stakeholders recognised gaining new knowledge; others got upset when stakeholders were ignorant. Stakeholders identified high attendance, being on time and sharing responsibilities as principles for a well-functioning group. Initially, time for regular monthly meetings was a challenge in the hospital groups and some stakeholders were frustrated when identified problems were judged not to be feasible to tackle.

#### Facilitation

Both the facilitator and mentor positions were advertised for, and thereafter recruitment followed. The facilitators recruited in the PeriKIP project shared several factors motivating them to become facilitators, including a wish to contribute to improving the health of women and children, changing and developing the situation in their area, and increasing awareness and knowledge of perinatal health among community members. In addition, facilitators also mentioned motivational factors relating to their situation, such as increasing their understanding of quality improvement and having a monthly salary.

The 2-week training of facilitators was considered sufficient. During the training, facilitators gained a relatively clear understanding of their role. Undertaking role-plays and field visits were challenging but essential parts of the training. Some of the facilitators wanted more practical training, including communication skills. The mentors received the same training as facilitators and, in addition, separate sessions about their specific tasks. The facilitator and mentor guides were perceived as useful, and the content was generally sufficient to fulfil their functions.

After the training, we now understand the role of facilitators, we understand why, how, and what facilitator means and of course understand our responsibilities. Facilitator, commune level

At the start of the project, the facilitators were tense, but they were more confident in their roles after a few meetings. They developed their role by updating themselves on the Internet and interacting with other facilitators and mentors. The distance was a barrier to interaction with others, and they wished for more regular opportunities to share experiences.

The study participants appreciated the facilitators. Facilitators had a dynamic role, varying with context and stakeholders. Study participants mentioned that facilitators asked stakeholders about perinatal health experiences from their villages, using non-technical terms, respectfully acknowledged participants’ contributions. They kept contact with community members, summarised meetings, took notes, kept track of members’ duties, encouraged and enthusiastically guided the discussions.

Our facilitator guided the discussions and helped out to collect results, gather report and identify new problems. Midwife, stakeholder group at commune level

The facilitators trained group participants in the PDSA method and provided support throughout the project. Study participants expressed that the facilitators supported the stakeholders in the quality improvement processes. This assistance involved problem-solving and encouragement but without making decisions for the group. The facilitator was seen as a person who understood the situation and supported the learning process. For example, the facilitators used the experience from one commune when helping another. The national guidelines for reproductive health [38] were introduced to the PeriKIP groups and used as sources of information when preparing for monthly meetings and selecting problems and actions.

Non-attendance required facilitators to act swiftly, contacting the missing stakeholder, stressing the importance of their contributions, updating the content they had missed, and sometimes involving the vice-chairperson or hospital head to motivate them to attend. When counteracting non-attendance, the assignment of the PeriKIP initiative was emphasised by higher health system levels.

The facilitators played an essential role in the PeriKIP project. They needed to be active and dynamic. At the commune level, the facilitators functioned well despite their lack of clinical experiences, but it was regarded as a necessity to have a professional health background at the hospital level. It was suggested that retired healthcare staff could be used as facilitators.

I think they [the facilitators] play an essential role in the meeting /…/ If we get stuck in selecting or prioritizing issues, they guide us, step-by-step. Women Union representative, stakeholder group at commune level

The mentors attended meetings to support the facilitators and kept in contact via mobile phones. The mentor provided support with clinical knowledge, through interaction with PeriKIP stakeholders, and in the PDSA process. Some mentors were however unsure if they were helping the facilitators in their role as they did not have the experience of facilitating local groups.

#### Context

There was a shortage of resources, both in the clinical practice and the facilitation process. Medicines, equipment and staff, e.g. obstetricians and paediatricians, were scarce. In the facilitation process, there was some lack of material for the facilitators. Furthermore, there were insufficient resources for travelling, material to attract people to meetings, suitable venues and communication materials, such as photos, videos, and illustrations.

In general, reaching people is a hard job. To meet us they would miss harvesting their corn. If we bring them something, it’d be smoother. Commune health center head, stakeholder group at commune level

Many conflicting traditions and norms existed in the study area. For example, men generally made decisions for women and were in charge of the family’s money. There were traditions related to pregnancy and childbirth with adverse health consequences for pregnant women and their newborns. In Cao Bang, many minority groups exist, and as with any group with long-standing and culturally anchored traditions, there was a reluctance to change. Another obstacle was hesitance among females belonging to ethnic minority groups to discuss reproductive health with male PeriKIP stakeholders. Some women in the PeriKIP groups considered travelling in remote areas dangerous, as there was a risk of being robbed.

There were geographic barriers to seeking care. The distance was also difficult for monthly meetings and implementing activities in remote areas. There were also language barriers to the communication with minority groups in Cao Bang.

### Outcomes

#### Relevance of problems and actions

There were 416 prioritised problems at the commune level, of which 96% were perceived as relevant, whilst at the hospital level, there were 88 prioritised problems, and all were perceived as relevant. Table 5 presents the most common problems.

**Table 5:** Prioritised problems in the PeriKIP groups

**Top-prioritised problems at the commune level (number of times prioritised across the 48 groups)**	**Top-prioritised problems at the hospital level (number of times prioritised across the four groups)**
▪ High rate of home delivery (34) ▪ Low awareness among pregnant women about self-care and newborn care (32) ▪ Pregnant women work hard during pregnancy (30) ▪ Women do not come for antenatal care according to national recommendations (29) ▪ Women avoid certain food after delivery (28) ▪ Poor nutrition among women during pregnancy (23) ▪ Smoking in the house where pregnant women and newly delivered mothers live (23) ▪ Poor preparedness for delivery (22) ▪ Difficult to detect pregnant women in the first 3 months (22)	▪ Lack of equipment (16) ▪ Lack of guidelines (7) ▪ Rooms for newborn care do not meet the hygiene standard (6) ▪ Midwives and nurses are not skillful in newborn resuscitation and essential newborn care (3) ▪ Poor counselling and support for immediate and exclusive breastfeeding (3) ▪ Lack of training or re-training on haemorrhage after delivery, mouth care and kangaroo mother care (3) ▪ Routine skin-to-skin contact after delivery is not practised (2) ▪ No screening for hepatitis B for pregnant women (2) ▪ Poor knowledge and improper use of drugs for preterm babies and newborns (2) ▪ Low coverage of hepatitis B vaccination for newborns within 24 h after delivery (2) ▪ Poor hand washing practices before delivery (2) ▪ Poor counselling on nutrition for women after caesarean Sect. (2)

The hospital groups worked with more problems than the commune groups (Table 6). Most problems identified on both levels were addressed by full PDSA cycles.

**Table 6:** Relevance of problems and actions

	**Commune-level PeriKIP group**	**Hospital-level PeriKIP group**	**Total**
Total number prioritised problems	416	88	504
Total number of relevant actions	1054	114	1168
Total number of prioritised problems being relevant, *n* (%)	401 (96)	88 (100)	489
Average number (*n*) of problems per unit	8.7	22	*Not relevant*
Relevant problems where the group only presented a plan of relevant actions (i.e. only made a *Plan*), *n* (%)	85 (8)	16 (14)	101 (9)
Relevant problems were the group presented a plan of relevant actions, undertook the actions and presented the result (i.e. undertook *Plan-Do and Study*), *n* (%)	182 (17)	14 (12)	196 (17)
Relevant problems were the group presented a plan of relevant actions, undertook the actions, presented the result and took new actions to achieve their goals (i.e. undertook *Plan-Do-Study and Act*), *n* (%)	787 (75)	84 (74)	871 (75)

#### Knowledge assessment and perspectives of gaining knowledge

Out of the 190 healthcare workers that completed the knowledge assessment before and after the intervention, there were 92 doctors, 55 midwives and 43 nurses. The respondents were working at commune health centres (*n* = 131) and hospitals (*n* = 59). Overall, the health workers increased their mean knowledge score from 10.8 before the intervention to 11.6 after the intervention (*p* < 0.05) with a maximum score of 22. Nurses increased their mean score by 1.2 (*p* < 0.05) and doctors by 0.8 (*p* < 0.05), whilst midwives increased their mean score with 0.4 (*p* = 0.19). Healthcare workers at the commune health centres increased their mean score by 0.7 (*p* < 0.05) and healthcare staff at hospitals by 0.9 (*p* < 0.05).

Gaining knowledge and insight was a category from the qualitative analysis. Recipients highlighted an increased need of knowledge among healthcare workers in a large number of areas, including various clinical skills, communication and understanding of the situation in the communes. Participating in the social innovation provided opportunities to learn from each other. Also, participation in PeriKIP made health workers realise the importance of their work and that change was possible.

I have learned new things about the healthcare for mothers and babies in the perinatal period. I now realize that before the program [PeriKIP] I did not fully understand the importance of medical and health care /…/. Now, as a person in charge of this field I think I still have to learn more and work harder so that I will lead and manage the health and medical care of the mothers and children better and more realistically. Vice-chair, stakeholder group at commune level

#### Observations of antenatal care

Four observations were conducted in each of the 48 communes before and after the social innovation. The mean score increased in seven out of eight domains with 0.3–4.3 (*p* < 0.05). Only the domain dealing with recording patient information did not change (Table 7).

**Table 7:** Findings from the observations of antenatal care

**Domain**	**Number of units**	**Number of items/score**	**Mean score difference (95% CI)**	***P*-value**^1^
Asking questions about the pregnant woman’s health status and reproductive health history	48	36	3.82 (2.65–5.00)	< 0.01
A general examination of the pregnant woman	48	7	0.89 (0.48–1.29)	< 0.01
Performing different tests	48	7	0.30 (0.09–0.51)	< 0.01
Tetanus vaccination (counselling and provision of vaccines)	48	2	0.56 (0.28–0.84)	< 0.01
Provision of essential drugs	48	5	1.75 (1.24–2.26)	< 0.01
Health education for pregnant women	48	4	0.35 (0.01–0.70)	< 0.05
Danger signs during pregnancy, birth preparedness and feedback on examinations	48	17	4.28 (3.08–5.49)	< 0.01
Recording patient information	48	3	0.00 (− 0.03–0.03)	1.00

^1^Based on *t*-test

## Discussion

We have described a social innovation where *facilitation* supported the implementation of systematic improvement cycles using PDSA through local stakeholder groups on primary, secondary and tertiary healthcare levels. It resulted in high attendance at locally-driven meetings, where 489 relevant problems were identified and 75% of these were addressed in full Plan-Do-Study-Act cycles to enhance perinatal health. The facilitators had significant roles in ensuring that groups with multiple stakeholders were established and functioned based on principles of mutual respect at all health system levels.

The PeriKIP social innovation was built on establishing a systematic identification of local problems, solutions and actions, as well as reflections on whether actions taken were beneficial and integrated and sustained in the health system. The foundation of this social innovation was that local ownership and governance were fundamentally important for a resilient health system that uses available resources effectively. Establishing this ownership required a structured approach and co-creation with key health system stakeholders. Through well-attended launching meetings, all involved levels were informed about plans, participated in forming the project and contributed to the organisation. In the establishment of the PeriKIP innovation, members of local systems occupied all key positions, i.e. researchers were not in focus. In the previous NeoKIP project, we successfully established local stakeholder groups on the primary level and reduced neonatal mortality substantially over three years [15]. However, even if NeoKIP was established with local ownership in mind [20] and had successful results even beyond the project period [21], integration into the health system with sustained activities did not happen [37]. The deficient ownership and integration in NeoKIP may be related to that involved researchers, both national and foreign, occupied crucial positions throughout the intervention period [37].

We applied facilitation to introduce and support the PDSA cycles at multiple levels of the healthcare system and in society. The stakeholder groups were inclusive, established through cross-hierarchical coalitions, where individuals representing different groups worked together to systematically improve the quality and utilisation of the services. The composition of the groups was essential to the successful results. Individuals with different areas of expertise were considered important pieces of the puzzle. However, including more stakeholders with influential positions in society could be an improvement. This social innovation was built on the NeoKIP experiences [15], and differed from the successful women’s group interventions [17], even if facilitation was used in all these efforts. The intention with PeriKIP was *not* to create new groups but to recognise and work within the current health system organisation and existing meeting structure with stakeholders at multiple levels, who already had health, welfare, and health services as their responsibility. Therefore, in comparison with other initiatives [15, 17], we believe PeriKIP processes and results have better opportunities to be sustained.

The PDSA cycles resemble an audit-feedback process, whereby practice and performance are measured and compared to standards or targets. The audit-feedback processes may lead to small but potentially significant improvements [46]. However, the outcomes depend on how feedback is given [46]. The application of trained facilitators in the initiation of these kinds of strategies is critical to ensuring an open climate with carefully moderated and constructive discussions. The traditional audit-feedback processes commonly start with an adverse outcome of a patient based on a death review or similar. In contrast, the PDSA cycle fundamentally builds on the group’s joint ownership of the problem. Targets and measures are developed from within, and improvement is commonly not a result of fault-finding on the individual level. It is not running the risk of becoming a blame game. The PDSA builds upon co-creation and departs from the recognition and involvement of stakeholders in the change process. The results show that the acceptability of this social innovation was good. Also, the facilitators’ adaptation to their new role was fast, which was not the case in the NeoKIP project [47]. This difference could be related to better training of facilitators as a consequence of previous evaluation from the NeoKIP, the publication of a facilitation guide [24] published by Harvey and Kitson that also contributed to informing the training alongside a stronger emphasis on achieving local ownership among stakeholders on all levels.

The adoption of the i-PARHIS framework to guide the intervention and the evaluation of the findings provided structure in this complex project. Although facilitation as a meta-strategy to achieve change is operationalised in the i-PARIHS framework, how to best design the training of facilitators remains unclear. There is a lack of guidance on how to ensure that facilitators are provided with adequate knowledge and skills to enact their roles. Although we appreciate the inclusion of the component recipients in i-PARIHS, our impression of that label is that it indicates a passive position. Rather, we see this component as target groups of innovations on different levels. In our case, the target groups were also actors and co-creators, concepts which could potentially also be a more fitting description than recipients.

The PeriKIP social innovation was only running for 12 and 18 months, respectively, at different levels of the health system. That might be sufficient for the stakeholder groups to learn and running the PDSA. However, a longer time for continuing to monitor what happened thereafter would have been preferable. We were also not able to study the potential that the social innovation might have on increasing the opportunity for enhanced connectedness between levels of the health system. For the process to be institutionalised, this innovation needs to be incorporated in the plans of the higher administrative structures of the health system. Social innovation is a cornerstone to achieve integrated people-centred health services, endorsed by the World Health Assembly in 2016. Our experience is that social innovation requires the identification of the right people to ‘sit around the table’ and an invested health system that can provide the larger structure that can encompass the integration of this way of working. Provided potential challenges in changing culture with regards to ‘who is an expert’ and current power dynamics and hierarchies, we believe these types of interventions would benefit from carefully recruited persons, facilitators, prepared to guide the development of the dynamic coalitions that social innovations constitute.

## Conclusions

Current global health goals will only be achieved through better health systems, which will rely on government and societal efforts. The 2030 agenda for women and children emphasises ending preventable deaths and promoting enabling environments for health and well-being. Despite a growing body of evidence on the impact of community engagement, maternal and child health services are often implemented without end-user involvement, resulting in a lack of trust, underutilisation, inequity and stagnating health indicators. The PeriKIP project, alongside a growing body of evidence on the impact of social innovations placing empowered communities in the front seat of their development, provides important evidence on how to strengthen the health system including strengthening the trust local community has in it as well as the inequities their skewed delivery is a consequence of. The PeriKIP social innovation was feasible to implement, acceptable among stakeholders across various healthcare levels in Vietnam, resulting in the identification of important problems and actions with plausibly favourable effects on perinatal health and survival. The innovation could be scaled up in the Vietnamese health systems, and could potentially be a relevant social innovation to test also in other settings. Our evaluation does however indicate that this process would not have happened if it would not have been for the facilitators playing a crucial role in establishing groups and enabling a constructive environment where all stakeholders’ opinions and suggestions are welcomed and where the participation of everyone is valued. The PeriKIP social innovation builds local capacity for implementation and could serve as a model where the implementation of evidence-based methods and programmes is understood, tailored and governed locally.

## Supplementary Information

**Additional file 1.** Logic model.

**Additional file 2.** Perinatal care knowledge assessment.

**Additional file 3.** Antenatal care observation form.

## Data Availability

In accordance with research ethics, the study participants were guaranteed anonymity. Thus, in the information provided prior to the consent procedure, potential participants were assured that the data would be available and managed only by the researchers. Thus, the complete datasets, such as transcripts from interviews, are not publicly available in full. However, essential excerpts of the transcripts and other collected data can be made available from the corresponding author upon reasonable request.

## Abbreviations

FGDs: Focus group discussions
i-PARIHS: Integrated-Promoting Action on Research Implementation in Health Services
NMR: Neonatal mortality rate
NeoKIP: Newborn Knowledge Into Practice
PeriKIP: Perinatal Knowledge-Into-Practice
PDSA: Plan-Do-Study-Act

## References

[1] Blencowe H, Vos T, Lee AC, Philips R, Lozano R, Alvarado MR (2013). Estimates of neonatal morbidities and disabilities at regional and global levels for 2010: introduction, methods overview, and relevant findings from the Global Burden of Disease study. Pediatr Res.

[2] Collaborators GBDD (2020). Global age-sex-specific fertility, mortality, healthy life expectancy (HALE), and population estimates in 204 countries and territories, 1950–2019: a comprehensive demographic analysis for the Global Burden of Disease Study 2019. Lancet.

[3] Bhutta ZA, Das JK, Bahl R, Lawn JE, Salam RA, Paul VK (2014). Can available interventions end preventable deaths in mothers, newborn babies, and stillbirths, and at what cost?. Lancet.

[4] Lawn JE, Blencowe H, Waiswa P, Amouzou A, Mathers C, Hogan D (2016). Stillbirths: rates, risk factors, and acceleration towards 2030. Lancet.

[5] Lawn JE, Blencowe H, Oza S, You D, Lee AC, Waiswa P (2014). Every Newborn: progress, priorities, and potential beyond survival. Lancet.

[6] Every Woman Every Child (2015). The global strategy for women’s, children’s and adolescents’ health (2016–2030).

[7] The Lancet Global H (2021). Progressing the investment case in maternal and child health. Lancet Glob Health.

[8] Ayob N, Teasdale S, Fagan K (2016). How social innovation 'came to be': tracing the evolution of a contested concept. J Soc Policy.

[9] van Niekerk L, Manderson L, Balabanova D (2021). The application of social innovation in healthcare: a scoping review. Infect Dis Poverty.

[10] Persson LA, Rahman A, Pena R, Perez W, Musafili A, Hoa DP (2017). Child survival revolutions revisited - lessons learned from Bangladesh, Nicaragua, Rwanda and Vietnam Acta. Paediatr.

[11] World Health Organization and International Initiative for Impact Evaluation. An evidence map of social, behavioural and community engagement interventions for reproductive, maternal, newborn and child health. Geneva: World Health Organization; 2017. Licence: CC BY-NC-SA 3.0 IGO.

[12] Black RE, Taylor CE, Arole S, Bang A, Bhutta ZA, Chowdhury AMR (2017). Comprehensive review of the evidence regarding the effectiveness of community-based primary health care in improving maternal, neonatal and child health: 8. summary and recommendations of the Expert Panel. J Glob Health.

[13] Berta W, Cranley L, Dearing JW, Dogherty EJ, Squires JE, Estabrooks CA (2015). Why (we think) facilitation works: insights from organizational learning theory. Implement Sci.

[14] Harvey G, Loftus-Hills A, Rycroft-Malone J, Titchen A, Kitson A, McCormack B (2002). Getting evidence into practice: the role and function of facilitation. J Adv Nurs.

[15] Persson L, Nga N, Målqvist M, Hoa D, Eriksson L, Wallin L (2013). Effect of facilitation of local maternal-and-newborn stakeholder groups on neonatal mortality: cluster-randomized controlled trial. PLoS Med.

[16] Malqvist M, Hoa DP, Persson LA, Ekholm SK (2015). Effect of facilitation of local stakeholder groups on equity in neonatal survival; results from the NeoKIP Trial in Northern Vietnam. PLoS ONE.

[17] Prost A, Colbourn T, Seward N, Azad K, Coomarasamy A, Copas A (2013). Women's groups practising participatory learning and action to improve maternal and newborn health in low-resource settings: a systematic review and meta-analysis. Lancet.

[18] Osrin D, Prost A (2010). Perinatal interventions and survival in resource-poor settings: which work, which don't, which have the jury out?. Arch Dis Child.

[19] Jokhio AH, Winter HR, Cheng KK (2005). An intervention involving traditional birth attendants and perinatal and maternal mortality in Pakistan. N Engl J Med.

[20] Wallin L, Malqvist M, Nga NT, Eriksson L, Persson LA, Hoa DP (2011). Implementing knowledge into practice for improved neonatal survival; a cluster-randomised, community-based trial in Quang Ninh province. Vietnam BMC Health Serv Res.

[21] Eriksson L, Nga NT, Hoa DTP, Duc DM, Bergstrom A, Wallin L (2018). Secular trend, seasonality and effects of a community-based intervention on neonatal mortality: follow-up of a cluster-randomised trial in Quang Ninh province, Vietnam. J Epidemiol Community Health.

[22] Knippenberg R, Lawn JE, Darmstadt GL, Begkoyian G, Fogstad H, Walelign N (2005). Systematic scaling up of neonatal care in countries. Lancet.

[23] World Health Organization (2014). WHO recommendation on community mobilization through facilitated participatory learning and action cycles with women’s groups for maternal and newborn health.

[24] Harvey G, Kitson AL, Ebooks Corporation. Implementing evidence-based practice in healthcare: a facilitation guide: Routledge, Taylor & Francis Group; 2015.

[25] Kitson A, Harvey G, McCormack B (1998). Enabling the implementation of evidence based practice: a conceptual framework. Qual Health Care.

[26] Dogherty EJ, Harrison MB, Graham ID (2010). Facilitation as a role and process in achieving evidence-based practice in nursing: a focused review of concept and meaning. Worldviews Evid Based Nurs.

[27] Thompson GN, Estabrooks CA, Degner LF (2006). Clarifying the concepts in knowledge transfer: a literature review. J Adv Nurs.

[28] Taylor MJ, McNicholas C, Nicolay C, Darzi A, Bell D, Reed JE (2014). Systematic review of the application of the plan-do-study-act method to improve quality in healthcare. BMJ Qual Saf.

[29] Greenhalgh T, Robert G, Macfarlane F, Bate P, Kyriakidou O (2004). Diffusion of innovations in service organizations: systematic review and recommendations. Milbank Q.

[30] Damschroder LJ, Aron DC, Keith RE, Kirsh SR, Alexander JA, Lowery JC (2009). Fostering implementation of health services research findings into practice: a consolidated framework for advancing implementation science. Implement Sci.

[31] Tuncalp, Were WM, MacLennan C, Oladapo OT, Gulmezoglu AM, Bahl R (2015). Quality of care for pregnant women and newborns-the WHO vision. BJOG.

[32] Chan G, Storey JD, Das MK, Sacks E, Johri M, Kabakian-Khasholian T (2020). Global research priorities for social, behavioural and community engagement interventions for maternal, newborn and child health. Health Res Policy Syst.

[33] General Statistics Office of Viet Nam (2015). Area, population and poulation density in 2013 by province 2013.

[34] World CE (2003). Health and ethinic minorities in Viet Nam. Technical series No. 1.

[35] Malqvist M, Nga NT, Eriksson L, Wallin L, Hoa DP, Persson LA (2011). Ethnic inequity in neonatal survival: a case-referent study in northern Vietnam. Acta Paediatr.

[36] Malqvist M, Eriksson L, Nguyen TN, Fagerland LI, Dinh PH, Wallin L (2008). Unreported births and deaths, a severe obstacle for improved neonatal survival in low-income countries; a population based study. BMC Int Health Hum Rights.

[37] Eriksson L, Bergstrom A, Hoa DTP, Nga NT, Eldh AC (2017). Sustainability of knowledge implementation in a low- and middle-income context: experiences from a facilitation project in Vietnam targeting maternal and neonatal health. PLoS ONE.

[38] Ministry of Health Vietnam. National standards and guidelines for reproductive health care services. Hanoi; 2009.

[39] Eriksson L, Huy TQ, Duc DM, Ekholm Selling K, Hoa DP, Thuy NT (2016). Process evaluation of a knowledge translation intervention using facilitation of local stakeholder groups to improve neonatal survival in the Quang Ninh province, Vietnam. Trials.

[40] Creswell JW, Plano Clark VL (2011). Designing and conducting mixed methods research.

[41] Moore GF, Audrey S, Barker M, Bond L, Bonell C, Hardeman W (2015). Process evaluation of complex interventions: Medical Research Council guidance. BMJ.

[42] Lewin S, Glenton C, Oxman AD (2009). Use of qualitative methods alongside randomised controlled trials of complex healthcare interventions: methodological study. BMJ.

[43] Linnan L, Steckler A (2002). Process evaluation for public health interventions and research.

[44] Harvey G, Kitson A (2016). PARIHS revisited: from heuristic to integrated framework for the successful implementation of knowledge into practice. Implement Sci.

[45] Elo S, Kyngas H (2008). The qualitative content analysis process. J Adv Nurs.

[46] Ivers N, Jamtvedt G, Flottorp S, Young JM, Odgaard-Jensen J, French SD (2012). Audit and feedback: effects on professional practice and healthcare outcomes. Cochrane Database Syst Rev.

[47] Eriksson L, Duc DM, Eldh AC, Vu PN, Tran QH, Malqvist M (2013). Lessons learned from stakeholders in a facilitation intervention targeting neonatal health in Quang Ninh province, Vietnam. BMC Pregnancy Childbirth.

