# Supplementary figures and images for: A facilitated social innovation: stakeholder groups using Plan-Do-Study-Act cycles for perinatal health across levels of the health system in Cao Bang province, Vietnam
Source: Implement Sci Commun. 2023 Mar 10;4:24. doi: 10.1186/s43058-023-00403-9 (PMC9999598; doi:10.1186/s43058-023-00403-9)

## PeriKIP logic model

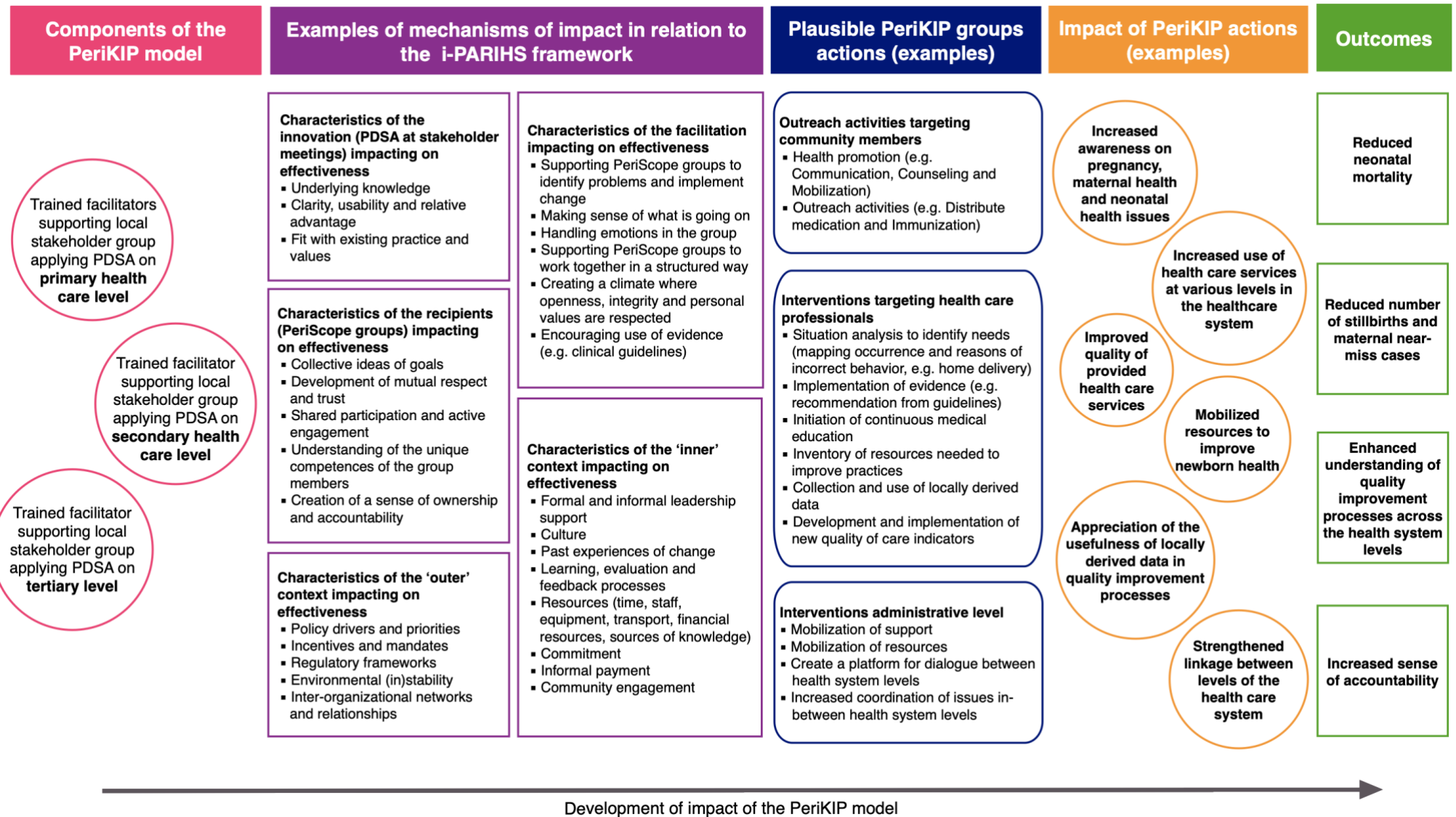

Supplement: Supplementary file 1 — Additional file 1. Logic model. [file 43058_2023_403_MOESM1_ESM.pdf]
