# Supplementary material for: A facilitated social innovation: stakeholder groups using Plan-Do-Study-Act cycles for perinatal health across levels of the health system in Cao Bang province, Vietnam
Source: Implement Sci Commun. 2023 Mar 10;4:24. doi: 10.1186/s43058-023-00403-9 (PMC9999598; doi:10.1186/s43058-023-00403-9)
Supplement: Supplementary file 2 — Additional file 2. Perinatal care knowledge assessment. [file 43058_2023_403_MOESM2_ESM.pdf]

PERINATAL CARE KNOWLEDGE ASSESSMENT

In the presented version below the correct answer is presented in ***bold****.*

Code: _______
Instructions: circle the letter corresponding to your answer

DEFINITIONS

1. **What period is perinatal period?**
    a. From the beginning to the end of the pregnancy
    b. From 22 weeks of the pregnancy to until delivery
    c. From 28 weeks of the pregnancy to until delivery
    d. **From 22 weeks of the pregnancy to 7 days after delivery**
    e. From 28 weeks of the pregnancy to 7 days after delivery

2. **Stillbirths are foetal deaths which occur:**
    a. Before 22 weeks gestational age
    b. **From 22 weeks gestational age until delivery**
    c. From 28 weeks gestational age until delivery
    d. From start of pregnancy until delivery
    e. From onset of labour until the delivery

ANTENATAL CARE

3. **During pregnancy, the woman should:**
    a. Come for ANC at least three times
    b. Come for ANC three times, ones in each trimester
    c. **Come for ANC at least three times, once in each trimester and if she has any problem**
    d. Come for ANC whenever she wants, regardless trimester
    e. Come for ANC only when she has a problem

4. **What type of information should be provided to a pregnant women during ANC?**
    a. During ANC the health worker must order the woman to come for facility-based delivery
    b. She will be informed about the sex of her foetus
    c. The woman should schedule for early ultrasound
    d. That vaginal examination will be undertaken at all ANC visits
    e. That breastfeeding should be initiated soon after delivery

5. **Intake of iron and folate supplement during pregnancy is advised:**
    a. For all pregnant women from the start of pregnancy until delivery
    b. For all pregnant women from the start of pregnancy until four weeks after delivery
    c. For all pregnant women from the start of pregnancy until six weeks after delivery
    d. Only advised for pregnant women who are anaemic from the start of pregnancy until delivery
    e. Only advised for pregnant women who are anaemic from the start of pregnancy until six weeks after delivery

6. **When counseling a pregnant woman about nutrition, be sure to:**
    a. Increase the number and amount of meals
    b. Double the number of meals
    c. Tell her to eat the same amount of food that she ate before her pregnancy
    d. Avoid eating fat food
    e. Avoid having a varied diet

7. **Maternal immunization against tetanus aims to:**
    a. Prevent tetanus for the pregnant woman
    b. Prevent tetanus for the neonate
    c. **Prevent tetanus for both the pregnant woman and the neonate**
    d. Prevent tetanus and other infections for the neonate
    e. Prevent tetanus and other infections for the pregnant woman

8. **What type of examination is should be undertaken at every ANC visit?**
    a. Undertake a vaginal examination at every visit
    b. Breast examination
    c. Height
    d. Measure abdominal circumference
    e. **Checking the woman's blood pressure at every visit**

9. **What type of test should be undertaken at every ANC visit?**
    a. Haemoglobin
    b. Test for syphilis
    c. Hepatitis
    d. HIV
    e. **Urine protein test**

POSTNATAL CARE

10. **When should the cord be cut following a normal delivery?**
    a. Immediately after the newborn is out
    b. After the placenta is out
    c. **After the cord stops pulsating or at 1-3 minutes after delivery**
    d. After 3 minutes after delivery
    e. When the baby starts crying

11. **What action should you take if a newborn is not breathing immediately after birth (after drying)?**
    a. Hold baby upside down
    b. **Clamp and cut cord and start ventilation**
    c. Start ventilation, no need to cutting cord
    d. Slap baby on buttocks
    e. Put baby skin-to-skin on the chest of mother

12. **At what rate should you ventilate a newborn using an Ambu bag during resuscitation?**
    a. 20-30 breaths per minute
    b. 30-40 breaths per minute
    c. **30-50 breaths per minute**
    d. 40-60 breaths per minute
    e. 50-60 breaths per minute

13. **In which order should the below actions be undertaken after delivery?**
    a. Dried; skin-to-skin contact; breastfeeding, clamp and cut cord; hepatitis B vaccine
    b. **Dried; skin-to-skin contact; clamp and cut cord; breastfeeding; hepatitis B vaccine**
    c. Dried; skin-to-skin contact; clamp and cut cord; hepatitis B vaccine; breastfeeding
    d. Dried; skin-to-skin contact; clamp and cut cord; weighing, measure length; breastfeeding
    e. Clamp and cut cord; dried; keep warm; breastfeeding; hepatitis B vaccine

14. **During the first hour following birth, the provider should**
    a. Measure the woman's blood pressure and pulse once, and insert a catheter to empty her bladder
    b. **Measure the woman's blood pressure and pulse and check the uterine tone every 15 minutes**
    c. If the mother is asleep do not measure blood pressure or pulse as rest is more important for her than that assessment
    d. Measure the woman's temperature and pulse, massage the uterus, and perform a vaginal examination to remove clots
    e. Measure the woman's blood pressure and pulse and check the uterine tone every 30 minutes

15. **When should breastfeeding be initiated?**
    a. When the baby cries
    b. Within 30 minutes after delivery
    c. After the first bath
    d. **Within one hour after delivery**
    e. When mother feel that there is milk

16. **A healthy newborn be kept in skin-to-skin position on the mothers chest after a normal delivery at least:**
    a. 15 minutes
    b. 30 minutes
    c. 60 minutes
    d. **90 minutes**
    e. 120 minutes

17. **Benefits achieved for the newborn by skin-to-skin contact include the following:**
    a. Keeps the baby warm and facilitates bonding
    b. Ready access to breastfeeding
    c. Less crying and reduced risk of infection
    d. **All of the above**
    e. Only alternative a) and b)

18. **Which medication or vaccine should give to a healthy newborn within the first 24 hours after birth?**
    a. Antibiotic for infection prevention and BCG vaccine
    b. **Vitamin K_1 and Hepatitis B vaccine**
    c. Vitamin K_1 and BCG vaccine
    d. Hepatitis B and BCG vaccine
    e. Not necessary to give any drug or vaccine within the first 24 hours

19. **How should the cord be cared for to prevent infections?**
    a. Clean daily using antiseptic fluid
    b. Cover the cord and tie a bandage or similar around the waist
    c. **Do not applying anything on the cord surface and keep the cord open**
    d. Cover the cord and tie a bandage or similar around the waist, change the bandage every day
    e. Apply antibiotic powder every second day

20. **How should the eyes of the newborn be cared for daily to prevent infection?**
    a. Apply antibiotic drop
    b. Apply NaCl 9‰ eye drop
    c. **Wash eyes only using clean water**
    d. Do nothing
    e. Apply eye antibiotic ointments

21. When should a newborn be examined?

a. Immediately after birth
b. Before discharge
c. Whenever the mother has concerns about newborn
d. **All of the above**
e. Only alternative a) and c)

22. Signs of infections in newborns include the following:

a. Crying
b. Fast breathing
c. Hypothermia
d. All of the above
e. **Only alternative b) and c)**

Code: _______

Van

Van, 26 years old is pregnant for the third time. She has one daughter 5 years old and second child is a boy 2 years old now. She has been admitted to a health facility today at 11.00 am with a complaint of labour pains since 4.00 am. She tells you that her membranes ruptured at 9.00 am.

Imagine that you are the nurse at the hospital and plot the following findings on the partograph.

1. **At 11.00 am:**
    a. Cervix is 4 cm dilated.
    b. She had 3 contractions every 10 min, each lasting less than 20 seconds.
    c. The FHR is 140 beats/minute.
    d. Membranes are absent, amniotic fluid is clear.
    e. Her pulse is 80 beats/minute, BP is 100/70 mm Hg and temperature is 37 deg Celsius

2. **11.30 am:** FHR- 130 b/min, contractions 3/10 each 35 sec, Pulse- 88/min, amniotic fluid clear

3. **Noon**: FHR- 136 b/min, contractions 3/10 each 40 sec, Pulse- 90/min, amniotic fluid clear

4. **12.30 pm:** FHR- 140 b/min, contractions 3/10 each 40 sec, Pulse- 88/min, amniotic fluid clear

5. **1.00 pm:** FHR- 130 b/min, contractions 3/10 each 40 sec, Pulse- 90/min, amniotic fluid clear

6. **1.30 pm:** FHR- 120 b/min, contractions 3/10 each 45 sec, Pulse-96/min, amniotic fluid clear

7. **2.00pm:** FHR- 118 b/min, contractions 3/10 each 45 sec, Pulse-96/min, amniotic fluid clear

8. **2.30 pm:** FHR- 112 b/min, contractions 3/10 each 45 sec, Pulse- 98/min, amniotic fluid meconium stained

9. **3.00 pm:** FHR-100 b/min, contractions 3/10 each 45 sec, Pulse- 100/min, amniotic fluid meconium stained, Cervix is 7 cm dilated, Temperature- 37.8 deg Celsius, BP-120/80 mm Hg

10. What action would you take at 3.00 pm?

Code: _______

Mai

Mai is 22 years old. She is pregnant for the first time. She has just completed eight months of pregnancy and had no problems so far. She has come to your health facility with her husband and mother-in-law. She appears anxious and is sweating profusely. She tells you that since morning she has vaginal bleeding, it is bright red and she does not have any pain.

1. What examinations will you conduct to assess Mai's condition? Which specific examination would you avoid conducting?

Code: _______

On examination you find the following: Mai appears pale and her skin is cold. Pulse 110/min, BP -90/60 mm Hg, Temperature 36.8 deg Celsius, Respiratory rate- 32/min, 1 vaginal pad soaked in last 5 minutes, FHR- 110 b/min, Presentation is vertex and longitudinal lie.

2. What do you think is the probable diagnosis?

3. How would you manage the case?

4. What advise would you give to Mai's husband?

PERINATAL CARE KNOWLEDGE ASSESSMENT

Code: _______

An

An is 24 years old. She is pregnant for the first time. She has never taken ANC. Looking at her you feel she must be about 36 weeks pregnant. Her husband and mother-in-law have brought her to your health facility because she was complaining of blurred vision and headache in the morning. They tell you that she had a convulsion on the way to the health facility.

1. What examinations will you conduct to assess An's condition?

Code: _______

On examination you find the following: An's Pulse 110/min, BP -160/100 mm Hg, Respiratory rate- 24/min, There is no pallor. On abdominal examination lie is found to be longitudinal and presentation is cephalic, FHR 110 b/min.

2. What do you think is the probable diagnosis?

3. What first line care would you provide before doctors help is received?
