# Supplementary material for: A facilitated social innovation: stakeholder groups using Plan-Do-Study-Act cycles for perinatal health across levels of the health system in Cao Bang province, Vietnam
Source: Implement Sci Commun. 2023 Mar 10;4:24. doi: 10.1186/s43058-023-00403-9 (PMC9999598; doi:10.1186/s43058-023-00403-9)
Supplement: Supplementary file 3 — Additional file 3. Antenatal care observation form. [file 43058_2023_403_MOESM3_ESM.pdf]

ANTENATAL CARE OBSERVATION FORM

**Information about observation**	
1. Name of observer:	3. Unit no
2. Date of observation: Day…………….month……………..Year:	4. Unit name

**Information about health provider**					
5. Health worker category	☐ Midwife	☐ Nurse	☐ Assistant doctor	☐ Doctor	
6. Sex of health worker	☐ Female	☐ Male	7. Consent given	Yes ☐	No ☐

No	Question	Yes	No	Don't know/can not observe
	**Health status of the woman**			
8	Did the health worker greet the client (and others present) in a friendly and respectful manner?	☐	☐	☐
9	Did the health worker introduce her/himself and title (midwife, nurse, etc.)	☐	☐	☐
10	Did the health worker call the client by her appropriate name or appropriate title?	☐	☐	☐
11	Did the health worker ask about or the client mention any of the following facts?			
A	Client's name	☐	☐	☐
B	Client's age	☐	☐	☐
C	Client's occupation	☐	☐	☐
D	Client's ethnicity	☐	☐	☐
E	Client's health status	☐	☐	☐
F	Client's disease history	☐	☐	☐
G	Client's current health condition	☐	☐	☐
I	HIV status	☐	☐	☐
12	Number of prior pregnancies (Observer: listen and record woman's number of prior pregnancies).<br>*If not asked leave blank*	No pregn. ___	Unknown no of previous preg ☐	No previous preg. ☐ →14
13	Did the health worker or client discuss any of the following complications for prior pregnancies?			
A	Heavy bleeding during or after delivery	☐	☐	☐
B	Anemia	☐	☐	☐
C	High blood pressure	☐	☐	☐
D	Convulsions	☐	☐	☐
E	Multiple pregnancies (twins or above)	☐	☐	☐
F	Prolonged labour	☐	☐	☐
G	C-section	☐	☐	☐
H	Assisted delivery (forceps, ventouse)	☐	☐	☐
I	Prior neonatal death (death of baby less than 1 month old)	☐	☐	☐
J	Prior stillbirth (baby born dead that does not breathe or cry)	☐	☐	☐
K	Prior abortion/miscarriage (loss of pregnancy)	☐	☐	☐
14	Did the health worker ask about or the client mention any of the following for current pregnancy?			
A	Date of the last menstrual period began	☐	☐	☐
B	Vaginal bleeding	☐	☐	☐
C	Fever	☐	☐	☐
D	Headaches or blurred vision	☐	☐	☐
E	Swollen face or hands	☐	☐	☐

F	Convulsions or loss of consciousness	☐	☐	☐
G	Severe difficulty breathing	☐	☐	☐
H	Persistent cough for 2 weeks or longer	☐	☐	☐
I	Severe abdominal pain	☐	☐	☐
J	Foul smelling discharge	☐	☐	☐
K	Frequent or painful urination	☐	☐	☐
L	Others (specify:			
M	Whether the client has suffered any violence	☐	☐	☐
N	If there are any other problems the client is concerned about	☐	☐	☐
	**General examination**			
15	Did the health worker wash his/her hands with soap or use alcohol hand rub prior to examination?	☐	☐	☐
16	Did the health worker perform any of the following general examination?			
A	Weigh the client	☐	☐	☐
B	Measure the height (for the first visit)	☐	☐	☐
C	Take the client's blood pressure	☐	☐	☐
D	Examine hands for edema	☐	☐	☐
E	Lung and heart examination	☐	☐	☐
F	Breast examination	☐	☐	☐
	**Testing**			
17	Did the health worker perform/counsel for following test?			
A	Urine test	☐	☐	☐
B	Hb	☐	☐	☐
C	Hematocrit	☐	☐	☐
D	Worm	☐	☐	☐
E	Counseling for voluntary HIV tests	☐	☐	☐
F	Perform or refer for a syphilis test	☐	☐	☐
G	Others (specify)	☐	☐	☐
	**TT vaccinations**			
18	Did the health worker provide the following immunization services:			
A	Prescribed or gave a tetanus toxoid (TT) injection	☐	☐	☐
B	Explained the purpose of the TT injection	☐	☐	☐
	**Providing essential drugs**			
19	Did the health worker prescribe/provide the following drugs:	☐	☐	☐
A	Prescribed iron or folic acid (IFA) or both	☐	☐	☐
B	Gave supply of iron or folic acid (IFA) or both	☐	☐	☐
C	Explained the purpose of iron or folic acid	☐	☐	☐
D	Explained how to take iron or folic acid pills/syrup	☐	☐	☐
E	Explained side effects of iron or folic acid	☐	☐	☐
	**Health education**			
20	Did the health worker:			
A	Inform the client about the development of the fetus?	☐	☐	☐
B	Discuss nutrition and healthy eating during pregnancy?	☐	☐	☐
C	Discuss about working and rest during pregnancy?	☐	☐	☐
D	Discuss any other particular issues in relation to health education (specify):	☐	☐	☐
21	Did the health worker counsel the client in any of the following reasons to seek immediate medical care?			
A	Seek immediate care if she has vaginal bleeding	☐	☐	☐

B	If she has convulsions	☐	☐	☐
C	If she has severe headaches with blurred vision	☐	☐	☐
D	If she has fever	☐	☐	☐
E	If she has severe abdominal pain	☐	☐	☐
F	If she has fast or difficult breathing	☐	☐	☐
I	Too weak to get out of bed			
22	Did the health worker counsel the client in any of the following ways about birth preparation?			
A	Asked the client where she will deliver	☐	☐	☐
B	Advised the client to prepare for delivery (e.g. set aside money, arrange for emergency transportation)	☐	☐	☐
C	If home delivery planned, advised the client to use a skilled health worker during delivery	☐	☐	☐
D	Discussed with client what items to have on hand at home for emergencies (e.g. clean delivery kit, sterile blade ect.)	☐	☐	☐
23	Did the health worker discuss breastfeeding?	☐	☐	☐
24	Did the health worker counsel on when to return for next visit?	☐	☐	☐
25	Did the health worker discuss family planning for use after delivery?	☐	☐	☐
26	Did the health worker ask whether the client had any questions?	☐	☐	☐
27	Did the health worker use any visual aids for health education or counseling during the consultation?	☐	☐	☐
28	Did the health worker speak using easy-to-understand language for the client?	☐	☐	☐
	**Recording patient information**			
29	Did the health worker look at the client's health card/booklet, either before beginning the consultation or while collecting information or examining the client (Observer: choose DK if no card/booklet)	☐	☐	☐
30	Did the health worker write on the client's health card?	☐	☐	☐
31	Did the health worker tell the client about the results of ANC examination?	☐	☐	☐
32	Record the time ANC consultation ended	………………………………		

Questions to the health worker (after observation)

33	Ask the health worker whether this is the client's first pregnancy	☐ Yes	☐ No	☐ Don't know	
34	Record the outcome of the consultation (what happened at the time the observation concluded)	☐ Client goes home	☐ Client referred (same facility)	☐ Client admitted (same facility)	☐ Don't Know
35	If client was admitted or referred, ask health worker what is client's diagnosis				

36	**Please comment on the quality of care provided**
